# Do DLX3 and CD271 Protect Human Keratinocytes from Squamous Tumor Development?

**DOI:** 10.3390/ijms20143541

**Published:** 2019-07-19

**Authors:** Elisabetta Palazzo, Alessandra Marconi, Carlo Pincelli, Maria I. Morasso

**Affiliations:** 1 Laboratory of Cutaneous Biology, Department of Surgical, Medical, Dental and Morphological Sciences, University of Modena and Reggio Emilia, 41100 Modena, Italy; 2 Laboratory of Skin Biology, National Institute of Arthritis and Musculoskeletal and Skin Diseases, National Institutes of Health, Bethesda, MD 20892, USA

**Keywords:** epidermal homeostasis, DLX3, CD271, SCC

## Abstract

Well-regulated epidermal homeostasis depends on the function of different classes of factors, such as transcription regulators and receptors. Alterations in this homeostatic balance may lead to the development of cutaneous squamous tumorigenesis. The homeobox transcription factor DLX3 is determinant for a p53-dependent regulation of epidermal differentiation and modulates skin carcinogenesis. The maintenance of skin homeostasis also involves the action of neurotrophins (NTs) and their receptors, Trk and CD271. While Trk receptor overexpression is a hallmark of cancer, there are conflicting data on CD271 expression and function in cutaneous SCC (cSCC). Previous studies have reported NT receptors expression in head and neck SSC (HNSCC). We show that CD271 is expressed at low levels in primary cSCC cells and the number of CD271+ cells correlates with cell cohesion in SCC spheroids. In normal epidermis, CD271 is expressed in proliferative progenitor cells and DLX3 in terminally differentiated keratinocytes. Brain-derived neurotrophic factor (BDNF) and neurotrophin 3 (NT3) increase DLX3 expression. In the absence of a functional BDNF receptor TrkB in keratinocytes, we hypothesize that the BDNF-dependent DLX3 response could be mediated via CD271. Altogether, our results support a putative CD271-DLX3 connection in keratinocytes, which might be crucial to preventing squamous skin cancer.

## 1. Introduction

Skin is the largest organ of the body, with a multitude of functions that depend on a well-regulated epidermal homeostasis. This homeostatic balance is dependent on the equilibrium between cells, mainly keratinocytes, and the function of transcription factors, receptors, growth factors, enzymes, and structural molecules [1]. Keratinocyte stem cells reside in the stratum basale and divide, yielding a limited number of early differentiated progenitor cells that continue to proliferate and then commit to differentiation. During the differentiation process, keratinocytes permanently exit the cell cycle, initiate expression of epidermal differentiation markers, and migrate supra-basally towards the surface of the epidermis. Any alteration of this fine balance may cause a pathological condition of the skin. In this context, cutaneous squamous cell carcinoma (cSCC) is the malignant prototype of altered epidermal homeostasis associated with uncontrolled proliferation, aberrant differentiation, and altered apoptosis.

cSCC represents the second most frequent type of skin cancer, comprising about 20% of skin malignancies and showing a rapidly increasing incidence worldwide [2]. cSCC constitutes a spectrum of progressively advanced malignancies, ranging from a precursor actinic keratosis (AK) to squamous cell carcinoma (SCC) in situ, invasive SCC and metastatic SCC. While differentiating, keratinocytes acquire a defined gene expression signature that includes cell cycle regulators and tumor suppressor genes [3]. Specifically, acquired mutations in the Ras or p53 genes in skin cells lead to altered response to growth factors and environmental insults, and consequently to keratinocyte hyperproliferation and neoplastic transformation [4,5]. However, the exact mechanism perturbing epidermal homeostasis and tipping the balance towards the development of cSCC, in contrast to other epidermal dysplasias, remains unclear. 

The DLX3 homeodomain regulator has been shown to be an important regulator of the calcium-dependent epidermal differentiation process [6]. By co-regulation with p53, DLX3 affects p53 downstream targets to modulate the cell cycle exit in the skin and acts as proliferative brake [7]. Loss of DLX3 is conductive to a pre-neoplastic state and its expression is missing in human and experimentally-induced cSCCs [7]. It has also been determined that absence of DLX3 function in skin leads to an inflammatory response [8], that involves activation of STAT3 signaling [9]. Therefore, the data support that DLX3-dependent pathways are necessary to prevent epidermal dysplasia and the activation of de-regulated inflammatory responses. 

In the skin, neurotrophins (NTs) and their receptors form a complex network of paracrine and autocrine loops with potential regulatory functions regulating epidermal homeostasis [10,11,12]. NTs are a family of growth factors, which includes NGF, BDNF, NT3 and NT4, whose activities are mediated by two classes of receptors: the common neurotrophin receptor CD271 (also called p75NTR) and the tyrosine kinases family of receptors (Trks). CD271 binds all NTs with equally low affinity, while Trks preferentially interact with a specific NTs [13]. CD271 can act as a Trk co-receptor to improve Trk affinity for NTs and Trk-mediated functions, or as a sortilin co-receptor for pro-NTs to promote cell death. On the other hand, CD271 comprises the so-called “death domain” and can signal apoptosis via its own signal transduction pathway by interacting with a number of downstream molecules [14]. 

In the epidermis, CD271 plays an important role in keratinocyte differentiation. CD271 characterizes a population of early differentiated keratinocytes and is implicated in the “switch” between stem cells and its progeny. Absence of CD271 function prevents keratinocyte differentiation and induces early differentiated keratinocytes to revert to stem cells [11]. CD271 mediates apoptosis in normal human keratinocytes and its overexpression restores susceptibility to cell death in normally resistant psoriatic keratinocytes [12]. These results strongly support the involvement of CD271 in the maintenance of epidermal homeostasis.

While the expression and function of DLX3 in SCC has been studied, there are few and contradictory data on the function of CD271 in epithelial cancer, specifically in SCC. Herein, we discuss the expression of NT receptors in squamous cancer and a possible feedback loop mechanism, by which DLX3 and CD271 might cooperate to maintain epidermal homeostasis and prevent cancer development. 

## 2. Results and Discussion

### 2.1. NT Receptors Are Heterogeneously Expressed in Squamous Cancer

Alteration of Trk receptor signaling pathways, including Trk protein overexpression, have been implicated in the pathogenesis of many cancer types, with *NTRK* gene fusions being the most well validated oncogenic events reported to date [15]. Moreover, pre-clinical and clinical evidence suggested their potential role as oncogenic drivers and predictive biomarkers for targeted inhibition, leading to the clinical development of a new class of compounds blocking the *NTRK* molecular pathway, which are currently under early clinical investigation [16,17]. Recently, a critical role has been suggested for the BDNF/TrkB system in cisplatin-resistance development in head and neck tumors, by utilizing Akt-dependent signaling pathways [18].

At the present time, there are conflicting data on the localization and function of CD271 in cutaneous SCC (cSCC). We have recently shown that CD271 is scarcely detectable in cSCC, while it has been preferentially detected in the basal layer of esophageal (ESCC) and oral SCC (OSCC) [19,20,21]. Recent work has shown that this receptor is functional in head and neck squamous cell carcinoma (HNSCC) and that inhibition of CD271 has profound negative effects on HNSCC’s tumor-initiating capacity [22].

**Figure 1:**
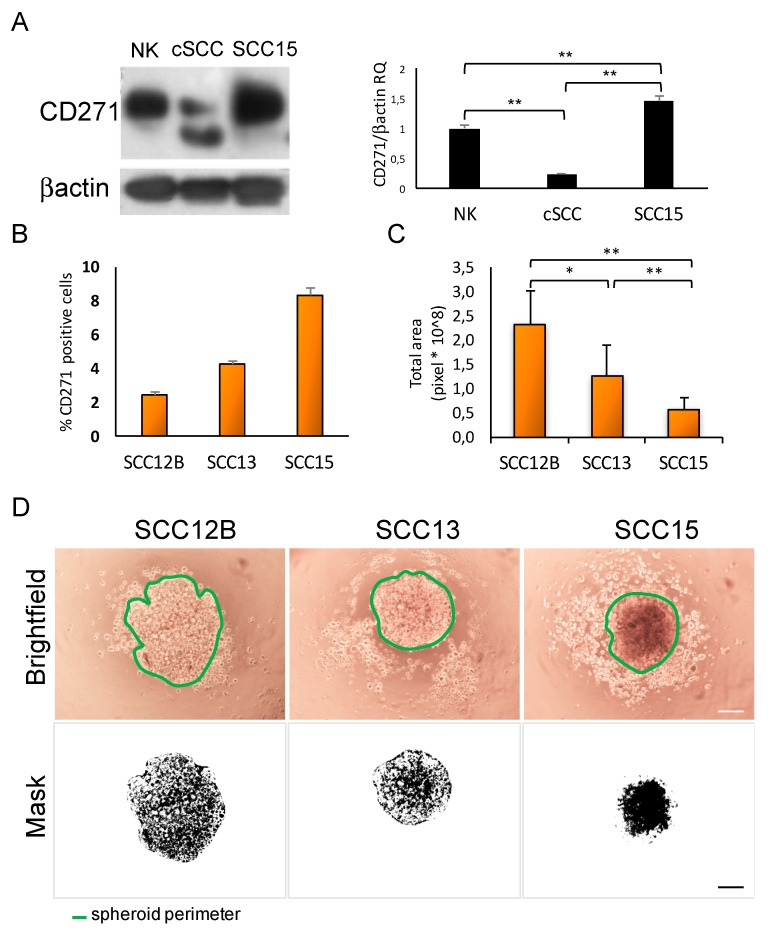
CD271 receptor expression in squamous cell carcimoma in vitro. (**A**) Left panel, CD271 expression in normal human keratinocytes (NK), keratinocytes from primary squamous cell carcinoma (SCC), and SCC15 cell line (SCC15) by Western blotting. Right panel, CD271/β-actin RQ (relative quantification) by densitometry analysis performed by ImageJ software. (**B**) Percentage of CD271 positive cells in SCC12B, SCC13, and SCC15 cell lines evaluated by FACS analysis. (**C**) Total area (or spheroid size) of SCC cell line derived spheroids, evaluated by ImageJ software analysis at 24 h after SCC cell plating. (**D**) Upper panel: representative images of SCC12B, SCC13, or SCC15 derived spheroids. Scale bar = 100 μm. Lower panel: SCC12B, SCC13, or SCC15 image masks generated from ImageJ software analysis and used to calculate the total area/size of each spheroid. For all results, data represent the mean from three independent experiments ± SEM. * for *p* < 0.05, ** for *p* < 0.01.

We analyzed the expression of CD271 in different samples of normal healthy keratinocytes (NK), primary cSCC, and in a mucosal SCC cell line (i.e., SCC15) (Figure 1A). Overall, our results show that CD271 is expressed at significantly lower levels in SCC cells from primary tumors compared to normal epidermal keratinocytes (NK). In contrast, CD271 expression is higher in the SCC15 cell line compared to primary tumors. The anti-CD271 antibody recognizes two bands by Western Blotting analysis, suggesting that CD271 might undergo post-translational processing in primary SCC cells (Figure 1A). This provides the possibility for a cancer-cell specific mechanism that potentially regulates CD271 expression and function. 

As alternative models for cSCC studies, we evaluated CD271 expression in different SCC lines, such as SCC12B, SCC13 and SCC15 (Figure 1B). SCC12B and SCC13 were originally obtained from SCC of the facial epidermis. SCC15 was derived from SCC of the tongue [23]. The percentage of CD271 expressing cells was substantially low in all cell lines, ranging from 2.5 to 8%. However, we observed a lower number of CD271 positive cells in cutaneous versus mucosal carcinoma. Furthermore, the highest percentage of CD271 correlates with a highest compactation in SCC spheroids. Indeed, the total area of SCC15 spheroids is significantly smaller when compared to SCC13 and SCC12B spheroid areas (Figure 1C), which display a less compact shape (Figure 1D). These observations are in agreement with the reduced cell-to-cell adhesion and increased area of CD271-silenced melanoma spheroids, and more compact shape displayed by melanoma spheroids overexpressing CD271 [24]. 

**Figure 2:**
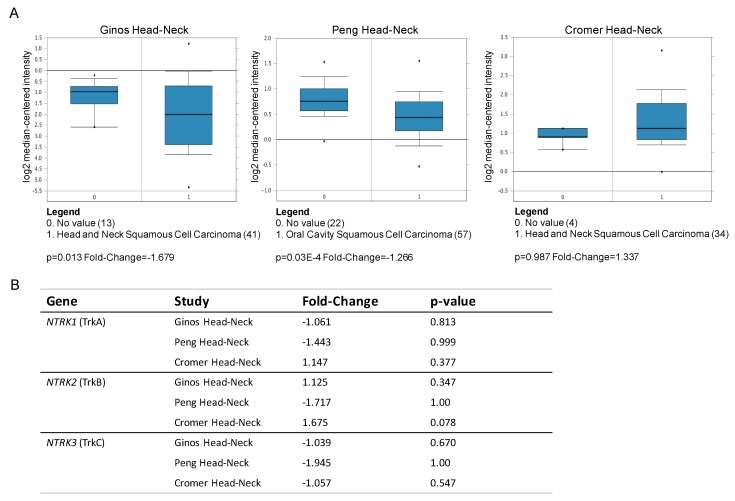
Neurotrophin (NT) receptor expression in vivo. (**A**) CD271 and (**B**) Trk receptors (TrkA, TrkB, TrkC) mRNA fold change from Oncomine (www.oncomine.org) microarray datasets for head and neck squamous cell carcinoma studies: Ginos head-neck (HNSCC vs normal; 54 samples), Peng head-neck (oral cavity SCC vs normal; 79 samples), and Cromer head-neck (HNSCC vs normal; 36 samples).

The analysis of public transcriptomic datasets from Oncomine (www.oncomine.org) determined there are no significant studies reporting cutaneous squamous carcinomas, while several studies evaluate HNSCC tumors versus normal tissue. We analyzed the expression levels of CD271 and Trk (TrkA, TrkB, TrkC) mRNA in the different datasets, such as the Ginos, Peng, and Cromer head-neck study. The analysis determined highly heterogeneous expression in HNSCC (Figure 2A,B). In the Peng head-neck study, CD271 expression is statistically down-regulated in tumors as compared to normal tissue. In addition, low levels of CD271 mRNA correlates with reduced expression of DLX3 and modulation of other tumor-associated markers [7].

### 2.2. DLX3-CD271 Feedback Loop in Human Healthy Keratinocytes

**Figure 3:**
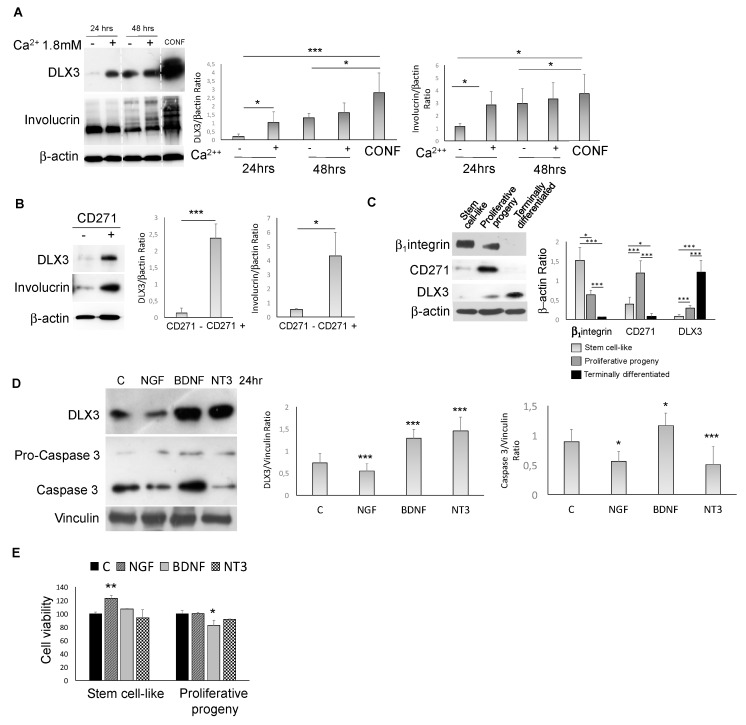
CD271-DLX3 interplay in keratinocytes (**A**) DLX3 and involucrin expression evaluated in human keratinocytes after 24 or 48 h of 1.8 mM calcium treatment or at confluence (CONF) or (**B**) in CD271 negative (CD271 -) and positive (CD271 +) cells. (**C**) β1-integrin, CD271, and DLX3 expression in keratinocyte subpopulations isolated as described in the Materials and Methods section. (**D**) Expression of DLX3 and caspase 3 in human keratinocytes treated with NGF (100 ng/mL), BDNF (100 ng/mL), or NT3 (100 ng/mL), detected by Western blot. p-values have been calculated as compared to control (C in the graph) for both DLX3 and caspase 3/β–actin ratio densitometry. (**E**) Cell viability of stem cell-like and proliferative progeny subpopulations evalutated by MTT assay at 48 h after the addition of NGF, BDNF, or NT3 at the previously indicated concentrations. p-values have been calculated as compared to control (C in the graph). For all results, data represent the mean from three independent experiments ± SEM. For all Western blotting, densitometry was performed by ImageJ software, as described in the Materials and Methods section. * for *p* < 0.05, ** for *p* < 0.01, or *** for *p* < 0.001.

Given the previously demonstrated role of DLX3 and CD271 in epidermal homeostasis [6,7,8,9], we checked for a functional connection between these factors in healthy keratinocytes. We found that DLX3 expression after calcium or confluence-induced keratinocyte differentiation overlaps with CD271 expression (Figure 3A). In particular, calcium was added to subconfluent keratinocytes in order to point out the effect of this pro-differentitative stimulus on proliferating and less differentiated keratinocytes. Furthermore, only the CD271+ sorted cells from total healthy normal keratinocytes (NK) express DLX3. These are the most differentiated cells, as evidenced by the expression of involucrin (Figure 3B). 

NK were separated according to β1-integrin expression as previously described [11], obtaining three keratinocyte subpopulations characterized by high clonogenic potential and stem cell-like properties, intermediate clonogenic potential with early progenitor features (proliferative progeny), and terminal differentiated phenotypes. Indeed, by colony-forming efficiency and long-term assays, plus the analysis of the epidermal differentiation markers, we have demonstrated that these cells represent distinct epidermal compartments [11]. However, given that β1-integrin is not the unique marker for keratinocyte stem cells, we used the term “stem cell-like” instead of “stem cells”.

CD271 was expressed by the proliferative progenitors, while DLX3 was expressed primarily in the most differentiated cells (Figure 3C). In addition, in NK, DLX3 expression was modulated by NTs (Figure 3D). In fact, recombinant nerve growth factor (NGF) promoted DLX3 downregulation, while brain-derived neurotrophic Factor (BDNF) and neurotrophin 3 (NT3) increased DLX3 expression. We have also shown that the apoptotic marker caspase 3 was activated in BDNF-treated cells and that BDNF is able to decrease cell viability of the proliferative progeny cells (Figure 3E). These data suggest that in absence of a functional BDNF receptor TrkB in NK [25], the BDNF-dependent DLX3 response could be mediated via CD271. These results are in line with the role of NGF in maintaining keratinocyte “stemness” [25]. On the other hand, DLX3 overexpression induced the upregulation of CD271 together with keratinocyte differentiation [7] (Table 1), indicating a potential DLX3-CD271 feedback loop in skin.

## 3. Conclusions

**Figure 4:**
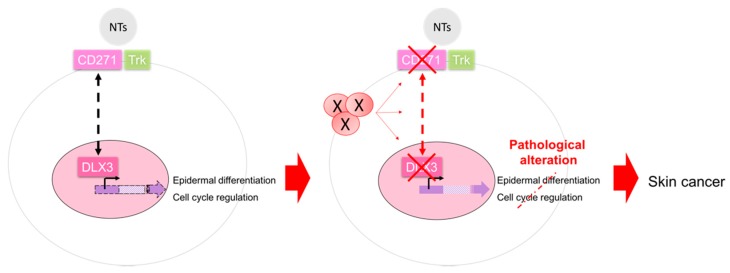
Alteration of CD271-DLX3 connection in keratinocytes. A CD271 and DLX3 feedback loop is required for the maintenance of the epidermal homeostasis through a fine regulation of the epidermal differentiation and cell cycle processes. The absence of their mutual signals, also due to other factors (X), is involved in pathological alterations that are a prelude to cancer development.

Altogether, our results support a putative CD271-DLX3 connection in keratinocytes and foment further studies to identify the signaling events that orchestrate their mutual regulation in the epidermis. DLX3 and CD271 are required for fine tuning the regulation of the epidermal differentiation process. Loss of expression or signaling alteration of either CD271 or DLX3-dependent pathways are critical for the development of epidermal cell dysplasia and, subsequently, squamous skin cancer (Figure 4).

## 4. Materials and Methods

### 4.1. Isolation of Primary Keratinocytes from cSCC Tissues

Primary keratinocytes from human cSCC were isolated as previously described [26]. Briefly, tumor samples from human cSCC patients were surgically removed and immediately stored in a sterile test tube containing medium and antibiotics. All tumor samples were collected with written informed consent of patients, according to the Declaration of Helsinki after approval of the Modena Medical Ethical Committee (protocol no. 184/10, 7 December 2010). Tumor tissues were washed with PBS without calcium and magnesium, cut into small fragments and digested in DMEM (Dulbecco’s modified eagle medium) containing 200 U/mL type I collagenase, 200 U/mL dispase and 70 U/mL DNase shaking for 2 h at 37 °C. The digested top tissue mixture was then filtered and centrifuged to collect the cells. Total cells were then seeded onto 3T3 feeder layers as previously described [27], and primary and secondary cell cultures were obtained. 

For Western blot analysis, collagen IV coated plates were prepared by seeding a human placenta-derived collagen IV solution (100 μg/mL, Sigma-Aldrich, St. Louis, MO, USA). Total cells from cSCC cultures, either at passage 0 or 1, were seeded on collagen IV pre-coated dishes in keratinocyte growth medium (KGM; Lonza, Walkersville, MD, USA) and maintained in culture until pre-confluent conditions. 

### 4.2. Isolation of Primary Keratinocytes from Healthy Skin and SCC Cell Line Cultures

Normal human keratinocytes were isolated from healthy skin biopsies obtained from waste materials from a surgical room. Patient consent for experiments was not required because Italian laws consider human tissue left over from surgery as discarded material. Isolated cells were cultured as described by Pincelli and colleagues [28]. Cells were maintained in culture with KGM until pre-confluence conditions and utilized for Western blot analysis. 

Stem cell-like, proliferative progeny, and terminally differentiated keratinocyte population enrichment was performed using the previously reported adhesion to type IV collagen method [11], which is based on the expression of β1-integrin. In detail, stem cell-like keratinocytes express the highest levels of β1-integrin when compared to proliferative progeny, while terminally differentiated keratinocytes do not express β1-integrin and do not adhere to collagen IV coated plates. Total human keratinocytes were left to adhere to collagen IV for 5 min in order to isolate the first population. Non-adherent cells from this plate were transferred to a different collagen IV coated plate and allowed to adhere overnight to obtain proliferative progenitors. Non-adherent cells represent the terminally differentiated cells and were removed from the adherent second population plate. All keratinocyte populations were maintained in KGM for 48 h after isolation and used for Western blot analysis. 

For calcium treatments, keratinocytes were grown in KGM until subconfluent conditions were reached and subsequently treated with 1.8 mM of calcium (Sigma-Aldrich, St. Louis, MO, USA) for 24 or 48 h, or confluence, prior being harvested for Western blot analysis. 

SCC12B, SCC13, and SCC15 cell lines were purchased from ATCC (Teddington, UK) and maintained in SCC medium (45% DMEM, 45% Ham’s F12, 10% fetal bovine serum, 2% L-Glutammine, 1% penicillin/streptomycin/amphotericin, 1M HEPES, and 400 ng/mL hydrocortisone). 

### 4.3. Keratinocyte Treatment with Neurotrophins and Transduction

Normal human keratinocytes were grown until 60% confluence in KGM medium. 24 h treatment with human recombinant neurotrophin nerve growth factor (NGF), brain-derived neurotrophic Factor (BDNF), and beurotrophin 3 (NT3) (all purchased from Sigma-Aldrich, St. Louis, MO, USA, and used at 100 ng/mL) was performed in KBM (keratinocyte basal Mmdium) plus bovine serum albumin (BSA) 0.1% (Sigma-Aldrich, St. Louis, MO, USA) and penicillin/streptomycin/amphotericin B (Lonza, Walkersville, MD, USA).

### 4.4. Multicellular Spheroids Culture

SCC spheroid formation was obtained by liquid overlay method [29]. Briefly, 96-well plates were coated with 100ul/well of 1.5% agarose (Sigma-Aldrich, St. Louis, MO, USA) diluted in DMEM:Ham’s F12 (Lonza, Walkersville, MD, USA) and sterilized by 45 min exposure to UVB radiation after agarose polymerization. Cells were seeded at a density of 3000/well and maintained in SCC medium. 24 h after plating, multicellular spheroids were visible with the microscope and photographed. Pictures were analyzed using ImageJ (Wayne Rasband, NIH, Bethesda, MD, USA) software.

### 4.5. Picture Analysis 

Pictures of SCC spheroids were analyzed using ImageJ software (Wayne Rasband, NIH, Bethesda, MD, USA) as previously indicated [30]. To calculate the areas, digital images were processed to 300 pixels/inch and converted to 8 bits. Then, the binary images were subjected to a “clean-up” procedure to eliminate artifacts and with the application “analyze particle”, the interested area was measured. The software provides a mask for each spheroid, which represents the analyzed particles, as indicated in Figure 1. The total area, which corresponds to the size of the spheroid, was calculated as the number of total pixels. Six spheroids for each cell line were analyzed and the experiment was performed in triplicate. 

### 4.6. Western Blotting

Total proteins from were extracted with RIPA (radioimmunoprecipitation assay) lysis buffer containing protease inhibitors. Equal amounts of protein for each sample were run on a 6%–18% SDS–PAGE gel and transferred onto a nitrocellulose membrane. Briefly, membranes were incubated overnight at 4 °C with the following primary antibodies: mouse anti-human CD271 (1:1000, Upstate Biotechnology Inc., Lake Placid, NY, USA), rabbit anti-human DLX3 (developed in Dr Morasso Lab [31]), mouse anti-human β1-integrin (1:500; Santa Cruz Biotechnology Inc., Santa Cruz, CA, USA), mouse monoclonal anti-human involucrin (1:1500; Sigma-Aldrich), mouse monoclonal anti-human β-actin (1:5000; Sigma-Aldrich), and mouse monoclonal anti-human vinculin (1:400; Sigma-Aldrich). After 3 washes with a PBS/tween solution, membranes were incubated with secondary antibodies, goat anti-mouse or goat anti-rabbit (1:3000; Bio-Rad Laboratories, Hercules, CA, USA), for 45 min at room temperature. Bands were visualized with a chemiluminescence detection system (Amersham Biosciences UK Limited, Little Chalfont Buckinghamshire, UK). Each experiment was performed in triplicate with different experimental samples. Each membrane was cut and hybridized for CD271, DLX3, involucrin, and β1-integrin antibodies or loading control antibodies (β-actin or vinculin). Densitometry analysis of the bands was performed by ImageJ software and data are presented as mean +/− SEM. 

### 4.7. Cell Sorting

To obtain CD271-positive cells, normal human keratinocytes were incubated in blocking buffer containing DMEM, 10% FBS, 0.1 M sodium azide, and 4% human gamma globulin (Sigma, St. Louis, MO) for 20 min on ice. After staining cells with 0.5% bovine serum albumin (BSA) in PBS with anti-CD271 antibody (1:100 in PBS, Lab Vision Corporation) at room temperature (RT) for 15 min, cells were resuspended with Alexa Fluor anti-mouse antibody (Invitrogen, Paisley, UK) in 0.5% BSA in PBS for 15 min at room temperature. Following this, cell viability solution (7-AAD solution, BD Via-Probe™, Biosciences Pharmingen, San Diego, CA, USA) was added to the pellet of cells for 15 min at room temperature. Finally, culture medium was added to cells. Negative control was obtained by omitting the primary antibody. Data were collected using a FACS Aria III flow cytometer (BD Biosciences) and analyzed on FACS Diva software (BD Biosciences). 

### 4.8. FACS Analysis

SCC12, SCC13, and SCC15 cells were incubated with anti-CD271 antibody (1:100 in PBS, Lab Vision Corporation, Thermo Fisher Scientific, Fremont, CA, USA) for 20 min at 4 °C, then were labeled with secondary antibody Alexa Fluor anti-mouse 488 (1:50, Thermo Fisher Scientific Inc, Waltham, MA, USA) for 20 min at 4°C. Cells were analyzed using an Epics XL flow cytometer (Beckman Coulter, Brea, CA, USA).

### 4.9. MTT Assay

5 × 10^3^ keratinocytes/well, of either stem cell-like or proliferative progeny cells, were seeded in 96-well culture plates in KGM. At 48 h from plating, cells were treated with human recombinant neurotrophin nerve growth factor (NGF), brain-derived neurotrophic factor (BDNF), and neurotrophin 3 (NT3) (all purchased from Sigma-Aldrich, St. Louis, MO, USA, and used at 100 ng/mL) in KBM (keratinocyte basal medium) plus bovine serum albumin (BSA) 0.1% (Sigma-Aldrich, St. Louis, MO, USA) and Penicillin/Streptomycin/Amphotericin B (Lonza, Walkersville, MD, USA). After 48 h, cells were incubated with 0.5% MTT (3-(4,5-dimethylthiazol-2-yl)-2,5-diphenyltetrazolium bromide) for 4h at 37°C and then dissolved with 100 μL isopropanol. The plate was read at 560 nm. Results are expressed as viability percentage, as compared to control.

### 4.10. Statistical Analysis

Statistical analysis was used for spheroids’ area comparisons and the relative quantification of Western blot bands. A Student’s *t*-test was perfomed by GraphPad Prism 7 (GraphPad Software, La Jolla, California, USA, www.graphpad.com). Significant p-values are indicated with * for *p* < 0.05, ** for *p* < 0.01, or *** for *p* < 0.001.

## Figures and Tables

**Table 1:** Neurotrophin (NT) receptors mRNA fold-change (microarray) in DLX3 overexpressing keratinocytes versus control cells reported by Palazzo et al. (2016) [7].

NT Receptors	Fold-Change
CD271 (*NGFR*)	3.19
TrkA (*NTRK1*)	1.19
TrkB (*NTRK2*)	−1.07
TrkC (*NTRK3*)	−1.2
